# Toward Better Conversations: Assessing Caregiver–Child Communication in Pediatric Oncology

**DOI:** 10.1007/s00520-026-10446-y

**Published:** 2026-02-17

**Authors:** Micah A. Skeens, Anna Olsavsky, Mariam Kochashvili, Nadeen Alshakhshir, Mays Basha, Amy R. Newman, Kathleen E. Montgomery

**Affiliations:** 1 The Abigail Wexner Research Institute at Nationwide Children’s Hospital, Columbus, OH USA; 2 The Ohio State University College of Medicine, Columbus, OH USA; 3 Division of Hematology/Oncology/BMT, Nationwide Children’s Hospital, 700 Children’s Drive, NEOB 3 Fl, Columbus, OH 43202 USA; 4 School of Nursing, University of Minnesota - Twin Cities, Minneapolis, MN USA; 5 Nell Hodgson Woodruff School of Nursing, Emory University, Atlanta, GA USA; 6 Marquette University, Milwaukee Wisconsin, USA; 7 Children’s Wisconsin, Milwaukee Wisconsin, USA; 8 Madison School of Nursing, University of Wisconsin, Madison, WI USA

**Keywords:** Pediatric cancer, Communication, Symptom science, Caregivers, Family functioning

## Abstract

**Purpose:**

Effective parent–child communication is central to coping with psychosocial challenges of pediatric cancer, yet few studies have examined how caregivers and children perceive their communication. This study investigated differences between caregiver and child reports of communication and associations with family relationship quality. We hypothesized children would report more open and positive communication than caregivers report, reflecting directional discrepancies in communication quality.

**Methods:**

Seventy-six caregiver–child dyads (N = 152) were recruited from two Midwestern pediatric hospitals. Children aged 8–17 with cancer and their caregivers independently completed measures of parent–child communication (PCCS) and family relationships (PROMIS). Descriptive statistics, correlations, and paired- and independent-samples t-tests examined differences and associations across dyads. Exploratory Actor–Partner Interdependence Models (APIM) investigated dyadic associations between child and caregiver communication and child family relationships.

**Results:**

Caregivers (10-item: M = 3.90, SD = 0.55; 20-item: M = 3.94, SD = 0.58) and children (M = 4.15, SD = 0.61) reported generally high-quality communication. However, significant differences emerged: children rated caregivers as more attentive listeners (t(74) = 2.53, p = .01, Cohen’s *d* = 0.29), emotionally open (t(74) = 2.30, p = .02, Cohen’s *d* = 0.27), and willing to discuss problems (t(74) = 2.86, p = .005, Cohen’s *d* = 0.33) than caregivers reported children. Across correlation and APIM analyses, child-reported communication was strongly associated with child-reported family relationships and caregiver-reported communication was strongly associated with caregiver-reported child family relationships (actor effects). Older caregiver and child age was linked to lower communication scores.

**Conclusions:**

Interdependent caregiver and child perceptions of communication represent an underrecognized factor influencing family functioning in pediatric cancer. Findings underscore the importance of routine communication assessment and highlight the need for developmentally tailored interventions.

## Introduction

Caregiver-child communication is a central mechanism through which families cope with the stress of pediatric cancer treatment and its psychosocial challenges [1]. Childhood cancer disrupts family routines and dynamics, placing emotional, logistical, and financial demands on both children and caregivers [2, 3]. However, communication is inherently dyadic and role-asymmetric: caregivers often serve as information gatekeepers [4] and decision-makers [5], while children communicate symptoms and preferences within developmentally constrained capacities [6]. Consequently, caregiver and child perceptions of “open communication” may diverge even when overall functioning appears strong.

Across pediatric oncology and other chronic pediatric conditions, open and supportive communication is associated with better psychological adjustment,, adherence, and family functioning [7–10]. Yet most research relies on single informant, limiting what can be learned about congruence or discrepancy within the dyad. Evidence from informant concordance research suggests that systematic differences may reflect developmental stage, family role expectations, and response tendencies (e.g., social desirability) rather than “accuracy” alone [11].

Developmental stage is especially salient in pediatric cancer: younger children often require simplified, reassuring explanations to manage fear and uncertainty, whereas adolescents seek autonomy and involvement in decision-making [12–14]. Thus, highlighting the need for tailored communication approaches that respect children’s evolving capacities while preserving family cohesion. These developmental and role differences may also shape responses to measures of communication (e.g., Parent–Child Communication Scale (PCCS)). For example, caregivers may be reluctant to endorse items implying negative child behavior (e.g., begin insulted when a child is angry), while children may rate caregiver listening or emotional openness more favorably.

This study addresses a current gap by examining both caregiver and child perspectives on communication in the context of pediatric cancer treatment. Specifically, it explores the degree of congruence and differences in their perceptions of these interactions. This study also explores how interdependent, dyadic perspectives of communication relate to dyadic perspectives of children’s family relationship quality. Based on role and developmental considerations, we hypothesized children would report more open and positive communication than caregivers would report. By clarifying how families experience and interpret communication, the study aims to inform the development of targeted strategies that support psychosocial adjustment, strengthen family functioning, and enhance the delivery of pediatric cancer care.

## Methods

### Procedures

Eligible caregiver–child dyads were recruited from two Midwestern children’s hospitals between October 2023 and March 2024. Participants were recruited during either outpatient oncology visits or inpatient chemotherapy admissions. Caregivers provided informed consent for both themselves and their child, while children gave verbal assent.

### Participants

Eligible children met the following criteria: 1) aged 8–17, 2) diagnosed with any form of cancer, 3) undergoing adjuvant cancer treatment for at least two months or had completed treatment within the past six months, and 4) able to read and understand English. Caregivers were eligible if they: 1) were 18 or older, 2) played a role in caring for the participating child, and 3) could read and understand English. Dyads were excluded if any condition prevented them from completing the research questionnaires, or if the child was only receiving radiation treatment for cancer. Only one caregiver was allowed to participate per child.

### Data Collection

Once consent was obtained, dyads had the option to complete the surveys during their visit or remotely. If they chose to complete surveys remotely, the study team sent a text or email containing a unique URL generated by REDCap. Each URL linked to the appropriate surveys for each participant (child or caregiver). Participants had 30 days (+ 3 business days) to complete the surveys independently. Upon successful completion, both the child and the caregiver received a $25 electronic gift card each, totaling $50 per dyad.

### Measures

#### Demographic and Clinical Characteristics

Caregivers reported on their own and their child’s demographic characteristics such as age, race, ethnicity, education, and income. Clinical characteristics of the children, such as diagnosis, disease and treatment status, were abstracted from the electronic medical records (EMR) by the study team.

#### Social Determinants of Health

Participants’ addresses were used to classify rural and Appalachian residency, as well as medically underserved area (MUA) status. Rural–Urban Commuting Area (RUCA) codes were used to categorize families as rural or non-rural. RUCA scores range from 1 to 10, based on population density, urbanization, and commuting patterns at the census tract and ZIP code levels. Scores from 4 to 10 were classified as rural, while scores from 1 to 3 were classified as non-rural [15]. Appalachian residency was determined based on the counties served by the Appalachian Regional Commission (ARC) in 2021 [16]. Participants residing in these counties were coded as 1 (Appalachian), and those residing outside these areas were coded as 0 (non-Appalachian). Medically Underserved Area (MUA) codes were used to assess access to primary care services [17]. Participants living in MUAs were coded as 1 (medically underserved), while those in non-MUA regions were coded as 0 (medically served).

#### Parent–child Communication (PCCS)

Child Version.

Children reported on their perceptions of their caregiver’s openness to communication. The child version of PCCS [18] included 10 questions, with responses rated on a 5-point Likert scale where 1 represented almost never and 5 almost always. Negatively worded items were reverse scored. Higher Mean scores indicated greater perceived openness to communication from caregivers and more frequent communication from the children. The Cronbach’s alpha of PCCS child version for this study was 0.73, which indicates acceptable internal consistency.

Caregiver Version.

Similarly, caregivers reported on their perceptions of their openness to communicate with their child and their children’s communication skills with them. This version of PCCS includes 20 items, with responses on a 5-point Likert scale where 1 represented almost never and 5 almost always. Negatively worded items were reverse scored. Higher Mean scores indicate greater perceived openness to communication by the caregivers and stronger communication skills in children. To align with child responses, we generated a 10-item mean score, but also report mean scores for all 20 items. The Cronbach’s alpha of PCCS caregiver version for this study was 0.89 for the 20-item version, and 0.74 for the 10-item version, which indicates acceptable internal consistency.

#### Family Relationship

Family relationships were assessed by both children and caregivers using PROMIS Pediatric Short Form v1.0—Family Relationships measure 8a [19]. This measure includes 8 items measuring responses on 1–5 Likert scale where 1 represented never and 5 represents always, with a 4 week recall period. In the U.S general population, the average score is 50 with a standard deviation of 10 with higher T-scores indicating a better family relationship. The child version measures child’s perceived family relationship (“I feel really important to my family”) and caregiver version assesses child’s family relationship from the caregiver perspective (“My child felt he/she was really important to our family”). Cronbach’s alpha of the child version of the measure for this study was 0.93 indicating excellent internal consistency. Cronbach’s alpha of the caregiver version of the measure for this study was 0.84 indicating good internal consistency.

### Statistical Analysis

Descriptive data were analyzed using IBM SPSS, version 28 for Windows and Actor–Partner Interdependence Models (APIMs) were analyzed using Mplus version 9. Descriptive statistics, including means, skewness, kurtosis, percentages and standard deviations, were calculated to summarize the sample and shape of the data. As all variables were deemed appropriate for parametric tests, Pearson correlations assessed associations among caregiver–child communication, child family relationships, caregiver age, and child age. Independent samples t-tests were conducted to explore differences in caregiver–child communication based on demographic characteristics. Paired samples t-tests were conducted to explore differences between child and caregiver for 10 matched PCCS items. APIMs were assessed to dyadically explore interdependent associations between communication and child family relationships among children and caregivers [20]. Children and caregivers were considered distinguishable based on their different family roles. Given the exploratory nature of these analyses, we opted to report saturated APIMs, which do not allow for the assessment of model fit as they are fully identified. Two APIMs were assessed: (a) communication as independent and child family relationships as dependent, and (b) child family relationships as independent and communication as dependent. Both models included covariates of caregiver and child age based on at least one significant association with either caregiver or child report of communication or family relationships.

## Results

### Demographic Characteristics

Seventy-six child–caregiver dyads (*N* = 152) participated. Most children and caregivers identified as White (children: *n* = 67, 88.2%; caregivers:* n* = 70, 92.1%) and non-Hispanic (children: *n* = 66, 86.8%; caregivers: *n* = 66, 86.8%). Female caregivers outnumbered male caregivers (*n* = 53, 69.7% vs. *n* = 22, 28.9%), and there were more male children (*n* = 49, 64.5%) compared to female children (*n* = 26, 34.2%). The average age of the children was 13.4 years (*SD* = 3.2). Most children were diagnosed with hematologic malignancies (*n* = 40, 52.6%) and had active disease status (presence of any disease on previous blood, bone marrow, or imaging) within 10 days prior to enrollment (*n* = 50, 65.8%) and had not reached remission. Most caregivers were biological parents of the child with cancer (*n* = 69, 88.5%) and with an average of three children per household (*SD* = 1.6). Most caregivers were employed (*n* = 49, 62.8%). Full demographic and clinical characteristics are detailed in Table 1.

**Table 1.** Demographics

Caregiver Demographic Characteristics		
**Caregiver’s Age (Mean, SD)**^**a**^		42.0 (7.3)
**Gender**	Male	22 (28.9%)
	Female	53 (69.7%)
	Missing	1 (1.3%)
**Relationship to the Child**	Biological Parent	69 (90.8%)
	Step-parent	2 (2.6%)
	Adoptive Parent	3 (3.9%)
	Grandparent	1 (1.3%)
	Other Caregiver	1 (1.3%)
**Number of Children in Household (Mean, SD)**		3.01 (1.6)
**Highest Grade of School Completed**	Less than High School	4 (5.3%)
	High School	12 (15.8%)
	Post High School (Technical or Trade School)	26 (34.2%)
	College	16 (21.1%)
	Graduate/Professional	16 (21.1%)
	Don’t know	1 (1.3%)
	Missing	1 (1.3%)
**Work Status**	Working now	49 (64.5%)
	Only temporarily laid off, sick leave, or maternity leave	5 (6.6%)
	Looking for work, unemployed	3 (3.9%)
	Retired	1 (1.3%)
	Disabled, permanently, or temporary	2 (2.6%)
	Keeping house	7 (9.2%)
	Student	1 (1.3%)
	Other	6 (7.9%)
	Missing	2 (2.6%)
**Rural Residency**	Rural	25 (32.9%)
	Non-Rural	51 (67.1%)
**Appalachian Residency**	Appalachian	8 (10.5%)
	Non-Appalachian	68 (89.5%)
**Annual Family Income**	Under $25,000	5 (6.6%)
	50,000 per year	9 (11.8%)
	75,000 per year	15 (19.7%)
	$75,001 −100,000 per year	10 (13.2%)
	150,000 per year	13 (17.1%)
	$150,001 or more	16 (21.1%)
	Prefer not to answer	6 (7.9%)
	Missing	2 (2.6%)
**Caregiver’s Race**	White	70 (92.1%)
	Black or African American	2 (2.6%)
	Asian	1 (1.3%)
	Some other race	3 (3.9%)
**Caregiver’s Ethnicity**	Not of Hispanic, Latino or Spanish origin	66 (86.8%)
	Mexican, Mexican–American, Chicano	7 (9.2%)
	Another Hispanic Latino, or Spanish origin	1 (1.3%)
	Missing	2 (2.6%)
*Child Demographic characteristics*		
**Child’s Age (Mean, SD)**		13.4 (3.2)
**Child’s Gender**	Male	49 (64.5%)
	Female	26 (34.2%)
	Missing	1 (1.3%)
**Child’s Race**	White	67 (88.2%)
	Black or African American	3 (3.9%)
	Asian	1 (1.3%)
	Some other race	3 (3.9%)
	Missing	1 (1.3%)
**Child’s Ethnicity**	Not of Hispanic, Latino or Spanish origin	66 (86.8%)
	Mexican, Mexican–American, Chicano	6 (7.9%)
	Another Hispanic Latino, or Spanish origin	2 (2.6%)
	Missing	2 (2.6%)
**Child’s Diagnosis**	Hematologic Malignancy	40 (52.6%)
	Non-CNS solid tumor	22 (28.9%)
	CNS tumor	14 (18.4%)
**Disease Status 10 Days Prior**	Documented Remission	24 (31.6%)
	Active Disease Presence of previous blood, BM, or imaging	50 (65.8%)
**Cancer Directed Therapy 10**	Yes	46 (59%)
**Days Prior**	No	30 (38.5%)

^a^Note: 5 caregivers had missing age values

On average, children reported higher quality communication (*M* = 4.14, *SD* = 0.61) than caregivers for both the 10-item (*M* = 3.90, *SD* = 0.55) and 20 item (*M* = 3.94, *SD* = 0.58) version of the caregiver PCCS, though both groups reported moderately high communication quality. Similarly, children reported higher average levels of family relationship quality (*M* = 54.44, *SD* = 9.86) than caregivers (*M* = 52.90, *SD* = 8.38), though both reported slightly higher than average (*M* = 50, *SD* = 10) child family relationship quality. There were low levels of skewness and kurtosis for child and caregiver reports of communication and family relationship quality (see Table 2). As it is more comparable to child reports, the 10-item version of the caregiver PCCS is used for all other analyses.

**Table 2:** Correlations

Measure	*N*	*M*	*SD*	Skew	Kurtosis	1	2	3	4	5
1. Child-Reported PCCS	75	4.15	0.61	−0.61	−0.30	-				
2. Caregiver-Reported PCCS (10 item)	76	3.94	0.58	−0.95	1.23	0.40** 95% CI: 0.19, 0.57	-			
3. Child-Reported Family Relationship	76	54.4	9.86	−0.85	−0.15	0.75** 95% CI: 0.63, 0.83	0.41** 95% CI: 0.20, 0.58	-		
4. Caregiver-Reported Family Relationship	76	52.9	8.38	−0.34	−0.56	0.27* 95% CI: 0.04, 0.46	0.53** 95% CI: 0.35, 0.68	0.35** 95% CI: 0.14, 0.53	-	
5. Caregiver Age	71	42.2	7.44	0.56	0.09	−0.29* 95% CI: −0.49, −0.06	−0.13 95% CI: −0.35, 0.11	−0.23 95% CI: −0.44, 0.004	−0.16 95% CI: −0.38, 0.07	-
6. Child Age	76	13.4	3.20	−0.18	−1.35	−0.02 95% CI: −0.25, 0.21	−0.24* 95% CI: −0.44, −0.02	−0.02 95% CI: −0.24, 0.21	−0.29** 95% CI: −0.48, −0.07	0.42** 95% CI: 0.21, 0.60

Note: PCCS stands for Parent–Child Communication Scale. **p < 0.01; *p < 0.05

Correlation analyses revealed that child-reported and caregiver-reported scores of caregiver–child communication were significantly correlated, *r*(75) = 0.40, *p* < 0.001. Child-reported caregiver–child communication was also significantly correlated with both child-reported family relationships, *r*(75) = 0.75, *p* < 0.001, and caregiver-reported family relationships, *r*(75) = 0.27, *p* < 0.05. Caregiver-reported caregiver–child communication was significantly correlated with child-reported family relationships, *r*(76) = 0.41, *p* < 0.001, as well as with caregiver-reported family relationships, *r*(76) = 0.53, *p* < 0.001. Caregiver age was significantly associated with child-reported caregiver–child communication, *r*(70) = −0.29, *p* < 0.05, indicating that children perceived lower quality communication as caregiver age increased. Similarly, child age was negatively correlated with caregiver-reported communication scores, *r*(76) = −0.24, *p* < 0.05, with caregivers reporting lower quality communication as children grew older. Caregiver age was not associated with caregiver-reported communication, similarly child age was not associated with child-reported communication (Table 2).

**Fig. 1:**
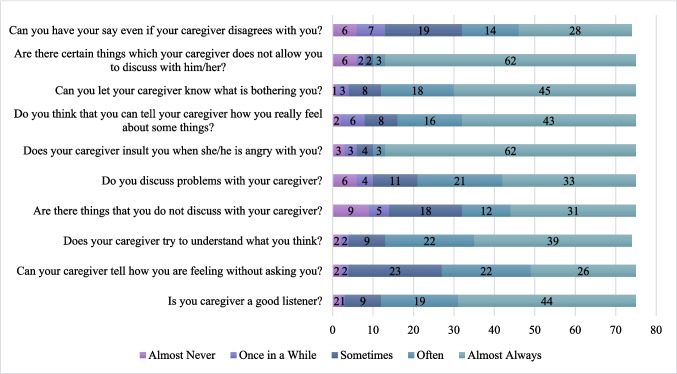
Child’s reports of caregiver-child communication. Note: Numbers on the bars represent the number of endorsements of each response

**Fig. 2:**
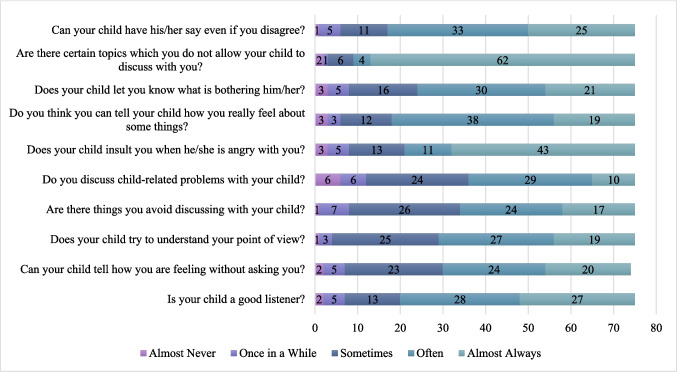
Caregiver’s reports of caregiver-child communication. Note: Numbers on the bars represent the number of endorsements of each response

**Fig. 3:**
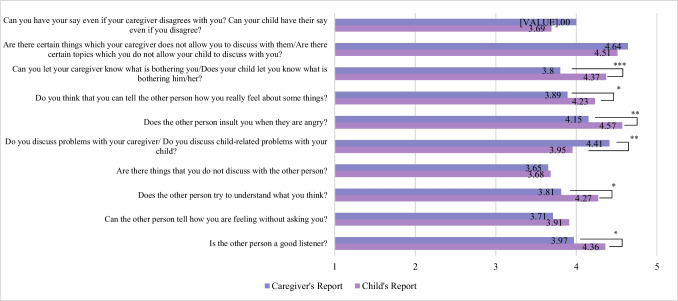
Mean child and caregiver’s report of caregiver-child communication. Note: ***p < 0.001; **p < 0.01; *p < 0.05

Both children (*M* = 4.14, *SD* = 0.61) (Fig. 1) and caregivers (10-item: *M* = 3.90, *SD* = 0.55; 20-item: *M* = 3.94, *SD* = 0.58) (Fig. 2) reported high overall quality of communication. Indeed, paired samples t-tests revealed several significant differences between child and caregiver perceptions across specific dimensions of communication (Fig. 3). On average, children reported significantly higher quality communication than caregivers (10-item caregiver PCCS: *t*(74) = 3.34, *p* = 0.001, Cohen’s *d* = 0.39; 20-item caregiver PCCS: *t*(74) = 2.82, *p* = 0.01, Cohen’s *d* = 0.33). As would be expected based on role and developmental stage, children rated their caregivers as better listeners (*M* = 4.36, *SD* = 0.94) than caregivers rated their children (*M* = 3.97, *SD* = 1.03; *t*(74) = 2.53, *p* = 0.01, Cohen’s *d* = 0.29). Similarly, children reported that their caregivers made more effort to understand what they were thinking (*M* = 4.27, *SD* = 0.97) than caregivers perceived their children made to understand them (*M* = 3.81, *SD* = 0.92; *t*(73) = 3.08, *p* = 0.003, Cohen’s *d* = 0.36). There was also a significant difference in how often problems were reportedly discussed. Children indicated that they talked about problems with their caregiver more frequently (*M* = 3.95, *SD* = 1.24) than caregivers reported discussing child-related problems with their child (*M* = 3.41, *SD* = 1.08; *t*(74) = 2.86, *p* = 0.005, Cohen’s *d* = 0.33), which may reflect appropriate roles. When asked about insulting behavior during anger, children reported that their caregivers insulted them more often (*M* = 4.57, *SD* = 1.04) than caregivers reported being insulted by their child (*M* = 4.15, *SD* = 1.17; *t*(74) = 2.81, *p* = 0.006, Cohen’s *d* = 0.32). Children also reported greater emotional openness, though this may be expected based on parent and child roles. They indicated a significantly higher ability to express their true feelings to their caregiver (*M* = 4.23, *SD* = 1.10) compared to caregivers’ reports of being able to express their feelings to their child (*M* = 3.89, *SD* = 0.97; *t*(74) = 2.30, *p* = 0.02, Cohen’s *d* = 0.27). Additionally, children rated themselves as significantly more able to let their caregiver know what is bothering them (*M* = 4.37, *SD* = 0.92) than caregivers believed their child could let them know what is bothering him/her (*M* = 3.80, *SD* = 1.05; *t*(75) = 4.30,* p* < 0.001, Cohen’s *d* = 0.49). Independent samples t-tests revealed no significant differences in child or caregiver communication (10-item versions) based on caregiver and child ethnicity, caregiver and child gender, caregiver and child race (White vs. non-White), rurality, MUA, and Appalachian residency.

### Exploratory Actor–Partner Interdependence Models

**Fig. 4:**
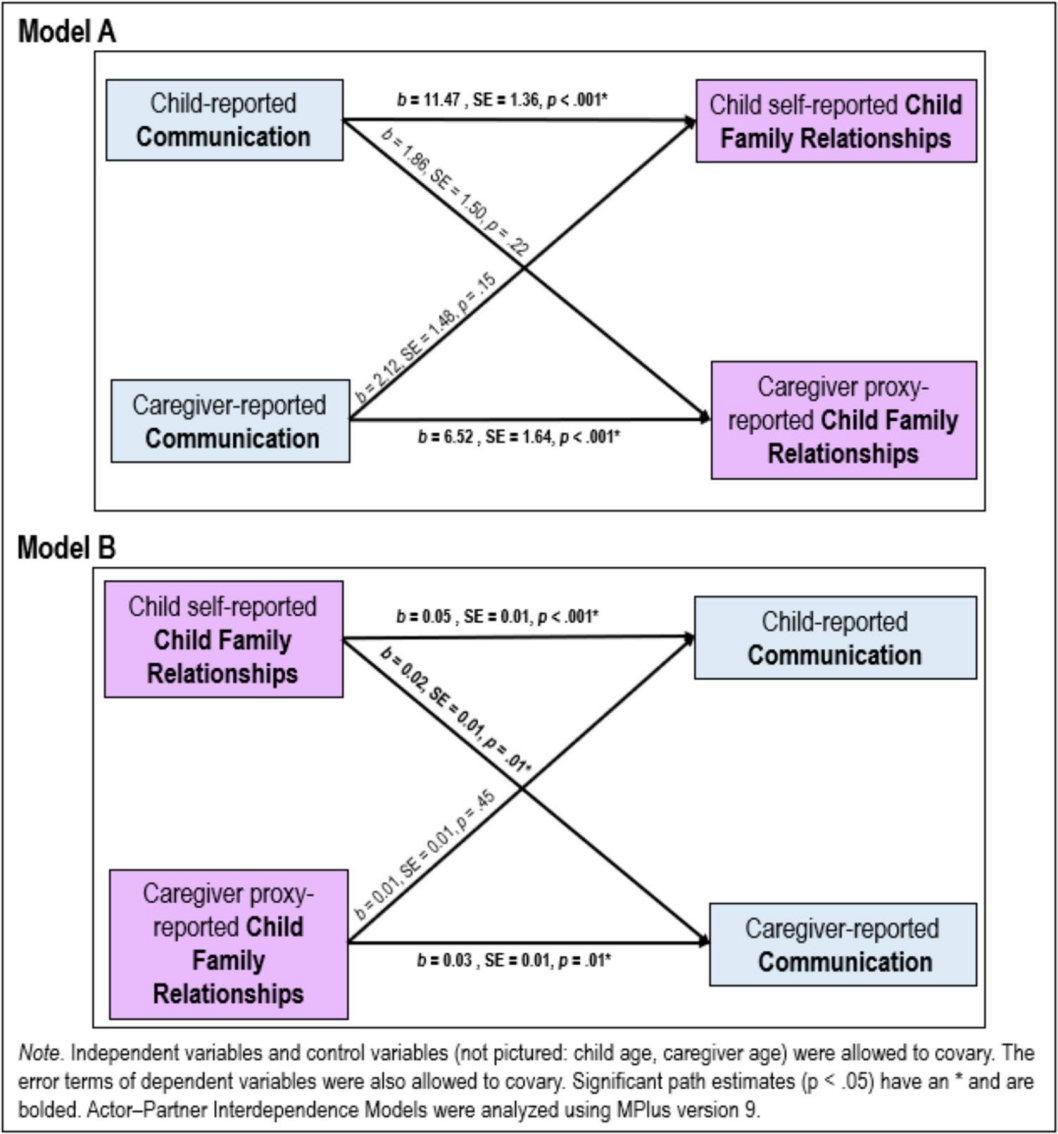
Exploratory Actor–Partner Interdependence Models

Finally, two APIM models were examined to explore dyadic associations between communication and child family relationships for caregivers and children (See Fig. 4). In APIM A, child and caregiver reports of communication were the independent variables, child and caregiver reports of child family relationships were the dependent variables, and child and caregiver age were included as covariates. In this model there were significant actor effects, defined as associations between one dyad member’s own independent and dependent variables; child communication was significantly and positively associated with child family relationship (*b* = 11.47, *SE* = 1.36, *p* < 0.001). Caregiver communication was also significantly and positively associated with caregiver proxy report of child family relationship (*b* = 6.52, *SE* = 1.64, *p* < 0.001). Neither partner effect, defined as associations between one dyad member’s independent variable with the other dyad member’s dependent variable, were significant (child communication to caregiver proxy-reported child family relationship:* b* = 1.86, *SE* = 1.50, *p* = 0.22; caregiver communication to child self-reported child family relationship: *b* = 2.12, *SE* = 1.48, *p* = 0.15). Caregiver and child age were not associated with child or caregiver reports of child family relationship. Child communication was associated with caregiver communication (*b* = 0.14, *SE* = 0.04, *p* = 0.001) and caregiver age (*b* = −1.53, *SE* = 0.57, *p* = 0.01), but was unrelated to child age. Caregiver communication was associated with child age (*b* = −0.42, *SE* = 0.21, *p* = 0.04), but was unrelated to caregiver age. Caregiver age and child age were associated (*b* = 10.00, *SE* = 3.01, *p* = 0.001).

In APIM B, child and caregiver reports of child family relationships were the independent variables, child and caregiver reports of communication were the dependent variables, and child and caregiver age were included as covariates. In this model, there were significant actor effects and one significant partner effect. Significant actor effects included a significant association between child self-reported family relationships and child communication (*b* = 0.05, *SE* = 0.01, *p* < 0.001) as well as between caregiver family relationships and caregiver communication (*b* = 0.03, *SE* = 0.01, *p* < 0.001). There was also a significant partner effect such that when children self-reported higher quality family relationships, caregivers reported higher quality communication (*b* = 0.02, *SE* = 0.01, *p* = 0.01). However, the other partner effect from caregiver proxy-reported child family relationships to child communication was not associated (*b* = 0.01, *SE* = 0.01, *p* = 0.45. Caregiver age was significantly associated with child communication (*b* = −0.02, *SE* = 0.01, *p* = 0.01), though no other covariates were related to child or caregiver communication. Child self-reported family relationship quality was associated with caregiver proxy-reported child family relationship quality (*b* = 28.56, *SE* = 9.91, *p* = 0.004), yet unrelated to child or caregiver age. Caregiver proxy-reported child family relationship quality was associated with child age (*b* = −7.65, *SE* = 3.16, *p* = 0.02), but was unrelated to caregiver age.

## Discussion

Differences emerged between caregiver and child perceptions of communication, highlighting an underrecognized factor influencing psychosocial outcomes in pediatric cancer care. Children reported higher levels of communication quality than caregivers recognized in them. However, alternative explanations for these discrepancies are plausible and warrant a cautious interpretation. Children may overestimate openness due to social desirability or a wish to present the family positively, and measurement features (e.g., different item pools across the child and caregiver PCCS versions and possible ceiling effects) may inflate apparent gaps. In addition, some items (e.g., discussing problems) may reflect normative role differences or, in some cases, role inversion rather than healthy openness. Thus, it is important to further investigate the origins and clinical meaning of caregiver-child perception gaps, given that both caregiver and child reports of communication quality were significantly associated with overall family relationship quality, underscoring the interdependence of communication and family functioning in the oncology context. This extends evidence that effective communication is not only protective but also relationally dynamic, whereby multiple domains of family well-being may be associated [9, 10]. Findings should be interpreted as preliminary, and generalizability is limited to mainly White, Midwestern, English-speaking families engaged in tertiary care.

Our findings also extend previous research documenting communication gaps in pediatric oncology. For example, Wiener et al. (2015) identified mismatches in caregiver and child understanding of emotional needs; our study expands this work by quantifying specific domains of discrepancy and linking them to family functioning outcomes. Consistent with earlier literature, open and supportive communication was associated with greater cohesion and reduced distress within families facing cancer [1]. Studies in other chronic pediatric conditions such as cystic fibrosis, type 1 diabetes, and juvenile idiopathic arthritis have similarly highlighted communication as a central determinant of adherence, emotional adjustment, and family cohesion [21, 22]. However, while most prior research has evaluated caregiver or child perspectives separately, relatively few have systematically examined congruence and discrepancy between the two. This study therefore adds important nuance by showing that even when families report generally “good” communication, misalignments in perception can still exist and these misalignments carry implications for family functioning.

Beyond overall discrepancies, developmental and cultural factors influenced perceptions of communication. Younger children and caregivers reported higher communication quality, while adolescents reflected the challenges of balancing autonomy with parental guidance. These developmental differences mirror prior research indicating that younger children are more reliant on parental reassurance and may respond positively to clear, consistent explanations [23]. In contrast, adolescents often seek greater independence in medical decision-making and may withhold information if they perceive parental control as limiting. This aligns with broader adolescent development literature showing that autonomy-supportive communication fosters resilience, whereas controlling communication can provoke resistance and conflict [24–26].

The findings have clear relevance for pediatric oncology practice. Oncologists and nurses are in a key position to coach families, by observing interactions during clinic visits and gently offering guidance. In line with this, the American Academy of Pediatrics and psychosocial oncology standards have called for evidence-based guidelines to improve communication in pediatric cancer care [27]. Therefore, routine assessment of family communication should be integrated into care through brief screening tools or structured discussions, allowing early identification of families struggling to maintain open dialogue. Communication-focused assessments have been recommended in psychosocial oncology guidelines [28] but are not consistently implemented. Our results support their integration as a standard component of pediatric cancer care. Second, interventions must be developmentally tailored. For younger children, caregivers may need support in providing clear, age-appropriate explanations and opportunities for expressive outlets such as play or drawing [29, 30]. For adolescents, clinicians should facilitate open conversations that respect autonomy, including opportunities for private consultations and involvement in treatment decisions. This echoes evidence that autonomy-supportive care strengthens adherence and emotional regulation in adolescent patients [31, 32]. Third, culturally responsive practices are essential. Studies have shown that culturally adapted interventions in pediatric chronic illness management improve adherence and family engagement [33]. In oncology, this may include interpreter services, linguistically appropriate educational materials, and collaborations with cultural liaisons or community health workers to ensure families feel empowered to participate in open dialogue. Such approaches are critical for reducing disparities and ensuring equitable psychosocial support. Together, these implications highlight that strengthening communication is not only a psychosocial priority but also a pathway to enhancing adherence, emotional well-being, and family resilience during treatment.

This study is not without limitations. The cross-sectional design prevents causal inference, limiting conclusions about how communication discrepancies influence outcomes over time. The study relied on self-reported perceptions, which may be subject to social desirability or recall bias. The child and caregiver PCCS versions also use different item pools and showed generally high scores in this sample, raising the possibility of ceiling effects and limited measurement equivalence that could exaggerate apparent discrepancies. Furthermore, 40% of the matched PCCS items used in the analysis were not identical statements. Although they were matched based on topic, caution must be taken when interpreting the presence of degree of incongruence. Additionally, the measure of communication included items for caregivers that may not actually indicate better quality communication with their child, despite contributing to better overall scores. For example, higher scores on the item “do you discuss child-related problems with your child?” and lower scores on the item “are there things you avoid discussing with your child?” were interpreted as higher quality communication. These items may instead tap into the concept of parentification, whereby a caregiver chooses to allow their children to assume an adult or parental role in the family [34]. Better measures of caregiver–child communication should be developed to avoid this issue.. These limitations underscore the need for replication in larger, more diverse samples and through multimethod approaches, including observational data. More broadly, the sample’s homogeneity (predominantly White, Midwestern, English-speaking families engaged in tertiary care) and unmeasured factors (e.g., language proficiency, socioeconomic status, immigration status, and clinic-level factors) may limit generalizability and confound observed associations. Finally, minimal clinically important differences (MCIDs) or other clinically meaningful thresholds have not yet been established for the Parent–Child Communication Scale (PCCS). Accordingly, the clinical significance of statistically significant differences—particularly those with small-to-moderate effect sizes—should be interpreted cautiously and in conjunction with distributional context and prior literature.

Because these data are cross-sectional, the observed associations cannot establish that systematic assessment or brief interventions will change caregiver–child communication or relationship quality over time. Rather, our findings are hypothesis-generating and support communication assessment as a way to identify dyads who may benefit from existing supports, which should be tested in longitudinal and intervention studies. “Future studies should explore the origins of caregiver–child perception gaps, investigating whether discrepancies arise from caregiver underestimation, child overestimation, or both. Longitudinal research is needed to examine how communication and perceptions evolve across the treatment trajectory, from diagnosis through survivorship, and to identify periods when interventions may be most impactful. Expanding work in underrepresented and non–English-speaking populations is essential to ensure findings are generalizable and to inform culturally tailored interventions. Finally, linking communication discrepancies directly to outcomes such as child anxiety, treatment adherence, and family functioning will provide stronger evidence for integrating communication-focused interventions into standard care.

## Conclusion

This study highlights significant discrepancies between caregiver and child perceptions of communication in the pediatric cancer context, with important implications for family functioning and psychosocial outcomes. While both groups reported generally high communication quality, children consistently rated communication more positively than their caregivers did. These mismatches, shaped in part by developmental and cultural factors, underscore the need for routine communication assessment and the development of targeted, developmentally appropriate, and culturally responsive interventions. Strengthening caregiver–child communication may serve as a critical pathway to enhancing psychosocial well-being and overall family resilience during pediatric cancer care.

## Data Availability

The datasets generated and analyzed during the current study are not publicly available but will be made available from the corresponding author on reasonable request.
